# An Interoperable Architecture for the Internet of COVID-19 Things (IoCT) Using Open Geospatial Standards—Case Study: Workplace Reopening

**DOI:** 10.3390/s21010050

**Published:** 2020-12-24

**Authors:** Steve H. L. Liang, Sara Saeedi, Soroush Ojagh, Sepehr Honarparvar, Sina Kiaei, Mahnoush Mohammadi Jahromi, Jeremy Squires

**Affiliations:** 1 Department of Geomatics Engineering, University of Calgary, Calgary, AB T2N1N4, Canada; soroush.ojagh@ucalgary.ca (S.O.); sepehr.honarparvar@ucalgary.ca (S.H.); sina.kiaei@ucalgary.ca (S.K.); mahnoush.jahromi@ucalgary.ca (M.M.J.); 2 SensorUp Inc., Calgary, AB T2L2K7, Canada; jeremy.squires@sensorup.com

**Keywords:** COVID-19, Internet of Things (IoT), interoperability, OGC SensorThings, OGC IndoorGML, contact tracing, physical distance detection, cough detection, spatial multi-criteria risk analysis, deep learning

## Abstract

To safely protect workplaces and the workforce during and after the COVID-19 pandemic, a scalable integrated sensing solution is required in order to offer real-time situational awareness and early warnings for decision-makers. However, an information-based solution for industry reopening is ineffective when the necessary operational information is locked up in disparate real-time data silos. There is a lot of ongoing effort to combat the COVID-19 pandemic using different combinations of low-cost, location-based contact tracing, and sensing technologies. These ad hoc Internet of Things (IoT) solutions for COVID-19 were developed using different data models and protocols without an interoperable way to interconnect these heterogeneous systems and exchange data on people and place interactions. This research aims to design and develop an interoperable Internet of COVID-19 Things (IoCT) architecture that is able to exchange, aggregate, and reuse disparate IoT sensor data sources in order for informed decisions to be made after understanding the real-time risks in workplaces based on person-to-place interactions. The IoCT architecture is based on the Sensor Web paradigm that connects various Things, Sensors, and Datastreams with an indoor geospatial data model. This paper presents a study of what, to the best of our knowledge, is the first real-world integrated implementation of the Open Geospatial Consortium (OGC) Sensor Web Enablement (SWE) and IndoorGML standards to calculate the risk of COVID-19 online using a workplace reopening case study. The proposed IoCT offers a new open standard-based information model, architecture, methodologies, and software tools that enable the interoperability of disparate COVID-19 monitoring systems with finer spatial-temporal granularity. A workplace cleaning use case was developed in order to demonstrate the capabilities of this proposed IoCT architecture. The implemented IoCT architecture included proximity-based contact tracing, people density sensors, a COVID-19 risky behavior monitoring system, and the contextual building geospatial data.

## 1. Introduction

Monitoring both “person-to-person” and “person-to-place” interactions is a critical issue for post COVID-19 reopenings [1,2]. Although person-to-person contact is a major factor of virus spread, recent studies have shown that a person can be infected even after the infected person has left the room [2,3]. When sharing the same indoor space, close contact can cause viruses to spread via air, objects, or floor, even after two to three days if the recommended protective equipment is not used, or disinfection is not carried out [4]. Geospatial information integrated into unified Internet of COVID-19 solutions plays an important role in monitoring the pattern of COVID-19 spread considering both infected “people” and “places”, and duration of contact. Such unified geospatial-enabled IoT solutions can be leveraged to understand the impact of virus spread for handling outbreaks, as well as, timely resource planning and allocation [5] on a cross-organizational scale [6,7,8,9].

There are many ad hoc Internet of COVID-19 solutions for combating the COVID-19 pandemic that use various sensor-based technologies [7,10,11,12,13,14]. An important way to evaluate and limit the spread of COVID-19 using the IoT is through the use of digital contact tracing solutions [14,15]. Digital contact tracing uses various combinations of close-range, proximity-based sensing technologies, such as smartphones, wearables [16], Bluetooth Low Energy (BLE) beacons [14], and positioning-based solutions [17] that use anonymous or randomly coded locations [11]. Regardless of the choice of technology, they all share the same goal: To identify and inform those who may have been exposed to the COVID-19 virus, or those who are in the high-risk category, so that they can take appropriate actions such as isolation, care, and treatment [18]. In addition to contact tracing apps, ongoing effort is being made to monitor post COVID-19 measures using the IoT [10,11,12,13]. However, these ad hoc IoT solutions are unable to interoperate with each other as they are developed using different sensors, data models, communication protocols, and applications without any interoperable way to interconnect these heterogeneous systems and exchange data. 

The major goal of this research is to design, implement, and evaluate an interoperable, standard-based, scalable IoT architecture for integrating the disparate Internet of COVID-19 Things (IoCT). This paper proposed an effective post COVID-19 information system for evaluating transmission risk for both people and places using disparate IoT systems, e.g., proximity-based beacons or Global Navigation Satellite System (GNSS)-based tracking, camera-based COVID-19 risky behavior detection, and contextual indoor geospatial information. A low-cost, multi-sensor, real-time IoCT was deployed that can be rapidly applied to different COVID-19 workplace reopening scenarios such as schools, office management systems, and smart cities. The proposed IoCT was employed to identify and limit the risk pattern of COVID-19 transmission especially within enclosed buildings. The risk of COVID-19 spread inside buildings from person-to-person and person-to-place interactions when taking into consideration different distances, durations, and types of activities (e.g., disinfecting activities) was modelled using the IndoorGML graph data model [19,20]. This research presents the innovative use of the Open Geospatial Consortium (*OGC*) [21] SensorThings Application Programming Interface (API) [22,23], as well as, the IndoorGML that uses Poincare duality to geo-reference IoT sensor observations for both 3D spaces and Node-Relation graphs in topology space. Our paper also argues that the integration of the IndoorGML and SensorThings API is critical for effective COVID-19 risk analysis and visualization. To the best of our knowledge, this paper is the first real-world implementation of the SensorThings API (*STA)* and IndoorGML. 

In order to validate the IoCT, an integrated COVID-19 solution was deployed and evaluated to monitor and analyze the risks of COVID-19 transmission in workplace reopening. For example, the following criteria may increase the risk of COVID-19 spread in an office room: If the room was used and the density of people was not regulated; if a sick person was present; or if people were not following social distancing rules etc. The proposed IoCT is able to access the risk history of each room using BLE proximity, deep learning-enabled cameras, and smart audio sensors. If the risk of spread in some rooms were high, appropriate alerts would be sent and received to shut down and disinfect the actionable list of contaminated places in order to prevent further transmission. This proposed IoCT was deployed using hybrid edge and cloud computing. The Calgary Centre for Innovative Technology (CCIT) building (with an area of 9530 m^2^) located in the University of Calgary campus [24] was used for a real-life testing scenario. The outcome of this solution will be useful for the protection of building staff and visitors as it integrates information-based solutions for real-time situational awareness and early warnings. The IoCT improves both the quality and speed of pandemic emergency response by enabling IoT system interoperability and unlocking necessary information for real-time decision making. The use of open-source software as well as the standard nature of this research boosts its usability as an international tool during the COVID-19 pandemic. 

In summary, the main contributions of this work are: (1) The innovative implementation of the SensorThings API and IndoorGML for analyzing indoor COVID-19 spreading risk patterns; (2) Deploying and validating a low-cost, standard-based, real-time IoCT for COVID-19 situational awareness that adheres to open IoT paradigms with interoperable agile access to individual COVID-19 sensor data; and (3) Evaluating person-to-place COVID-19 workplace reopening scenarios for the first time using an open geospatial-based IoT. 

The remainder of this paper is organized as follows: Section 2 presents background information on IoCT conceptual modelling using new trends in geospatial open standards; Section 3 presents the architecture proposal for the IoCT platform; Section 4 details the proof of concept of our architecture proposal using a workplace reopening scenario; Section 5 discusses the experimental results of the IoCT with the use of various sensors; and finally, this paper finishes with conclusions and an overview of future work in Section 6.

## 2. Background

### 2.1. Person-to-Place Interactions in Post COVID-19 Workplace Reopening Scenarios

The IoCT offers various use cases based on the digital monitoring of the physical world and humans using smart sensors that collect and deliver information. For this paper, we explored different COVID-19 transmission risks in miscellaneous workplace reopening scenarios. “Close contact” with a COVID-19 patient, which mostly occurs indoors, was one of the most common methods of COVID-19 transmission [4,25]. Based on our literature review, COVID-19 “close contact” was defined as contact occurring within a period of time longer than 15 min, and a physical distance of less than 2 metres in cases of face-to-face interaction [25]. When sharing the same space, viruses can spread via air, objects, or floor even after two to three days if protective equipment is not used, or disinfection carried out [4]. A digital timely proximity tracing system can effectively limit the spread of contagious diseases by collecting information about people or places that an individual (with confirmed or suspected infection) may have had close contact with or had been to [14]. If a person with COVID-19 was in close proximity to a place, those affected locations (e.g., businesses, public sites, or buses) would then be considered contaminated places (geospatial features). This geospatial information can be used to help in closing, disinfecting, alerting, and defining appropriate safe paths and neighborhoods. For example, if a location is popular with families or the elderly, additional facilities and organizations in the area may need to be alerted to possible exposure in their neighborhood. Health workers will receive timely and relevant alerts to close off and disinfect the actionable list of contaminated places to prevent further transmission. Moreover, organizations at each location can be provided with forms to send to their staff, customers, or visitors informing them of possible contamination, and requesting that they complete a contact trace survey for health authorities.

**Figure 1:**
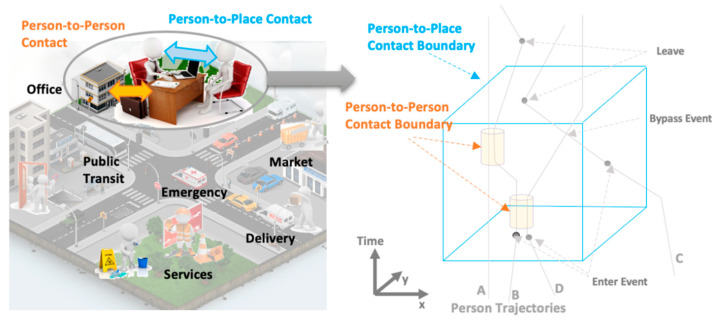
Scenarios for *COVID-19* virus spread in workplace reopening: The *Person-to-Person* and *Person-to-Place* contact (**left**). Spatiotemporal representation of *person-to-person* contact boundaries is shown using two orange cylinders (**right**); spatiotemporal representation of *person-to-place* contact boundary in a room is shown using a blue cubic (**right**); and spatiotemporal representation of a room, people trajectories are shown as gray lines.

Three important questions in COVID-19 risk evaluation are: “Who was in close contact with the COVID-19 positive person?” and “What places did the COVID-19 positive person visit, and who else visited those places after that?” The first question can be automatically queried using person-to-person contact tracing. The second question addresses the main focus of this paper which is person-to-place contact tracing. If a COVID-19 positive person was in close proximity to a place, those affected locations (geospatial features such as businesses, or public sites, or buses) are considered to be potentially contaminated places. This information is useful for advising people on safety measures, self-quarantine, and for issuing cleaning alerts. As shown in Figure 1, both people and places, as well as duration of contact, play an important role in the pattern for COVID-19 spread. 

The primary step when assessing the COVID-19 virus transmission scenarios is determining the factors affecting the risk of COVID-19 spread. According to [26], COVID-19 risk prevention and control depend on population flow. For this research, the epidemic risk status is assessed based on three levels of personnel flow strategies: (1) Staying at home, temperature monitoring, and traffic control; (2) Wearing a mask and restricting gatherings; and (3) Strengthening health management. Additionally, based on existing studies from both the World Health Organization (WHO) [27] and the Government of Canada website [28], the number of active virus particles in a place is considered to be the most critical factor in determining the risk of infection. Virus particles live for different lengths of time which vary depending on several factors, especially with regards to the composition of different surfaces. To identify and limit the risk pattern of COVID-19, a range of use cases can be considered that utilize the interactions of people, places, and available sensor information for a workplace. The following table (Table 1) sums up the office cleaning use case using person-to-place scenarios for post COVID-19 workplace reopening. 

### 2.2. Interoperability Using the OGC SensorThings API

Interoperability is a major challenge for the IoT. The real potential of IoT lies in the “systems of IoT systems” rather than with disparate IoT silos [29,30]. An interoperable IoT system of systems provides a uniform way for sharing, finding, and accessing IoT sensing and tasking capabilities, the Internet, and different applications [31]. Interoperability requires layers of standards in order to address the heterogeneity issues amongst sensors, data, and networks [32]. Data and sensor interoperability refer to the ability to exchange and understand data formats, protocols, and sensor models. Network interoperability has no value if the bits and bytes are delivered but cannot be interpreted, i.e., if the data being exchanged over the network cannot be understood by machines a priori. Further, various levels of interoperability include synthetic, semantic, and cross-domain interoperability which mean the standardization of conceptual models, practices, and policies from disparate systems. 

The OGC SensorThings API (OGC STA) [22,25] is an OGC and United Nation’s International Telecommunication Union Telecommunication (ITU-T) standard that defines a data model and an API for IoT sensing and tasking interoperability. The OGC STA is part of the well-established OGC Sensor Web Enablement (SWE) suite of open international standards [23]. SWE standards are in use by many large-scale organizations such as the Department of Homeland Security (DHS) [33], National Aeronautics and Space Administration (NASA), National Oceanic and Atmospheric Administration (NOAA), United States Geological Survey (USGS) [34], Natural Resource Canada (NRC), the World Meteorological Organization (WMO), and many others, including private sector companies [29,35,36]. The previous generation of SWE standards, such as the Sensor Observation Service (SOS), are heavyweight when it comes to running applications in edge devices with limited resources [37]. OGC STA represents the new generation of SWE standards that was specifically designed for IoT applications and is thus efficient and lightweight, e.g., it uses the REpresentational State Transfer pattern (RESTful) and the efficient JSON encoding. The *OGC* SensorThings API follows the ODATA (Open Data Protocol) for managing the sensing resources. As a result, it has a REST-like API and supports the Hypertext Transfer Protocol (HTTP) create, read, update, and delete operations (i.e., GET, POST, PATCH, DELETE) and ODATA query options (select, expand, filter, orderby, top, skip) for data retrieval [38]. In addition to supporting HTTP, the *OGC* SensorThings API has an extension for supporting Message Queuing Telemetry Transport (MQTT) for the creation and real-time retrieval of sensor Observations [39]. 

**Figure 2:**
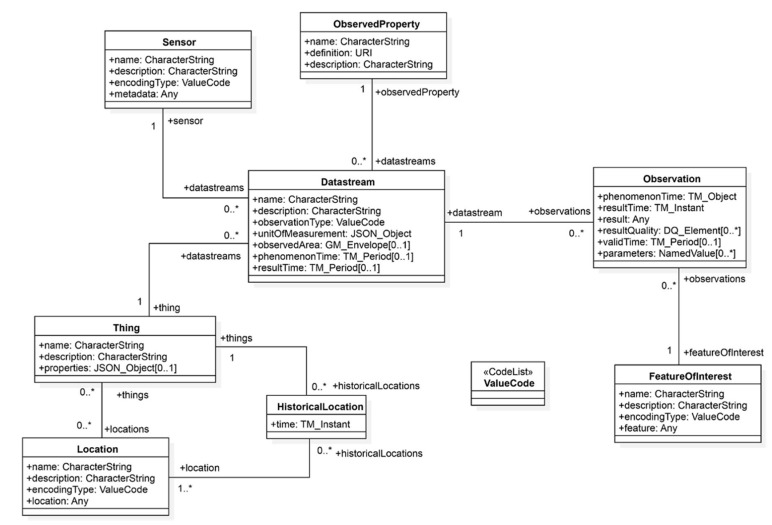
*OGC STA* Sensing Entities Core Data Model [40]: in this figure, “*” denotes “many” instances in ‘0 to many” and “1 to many” relationship.

The OGC STA enables interoperability for two layers: (1) Service interface, and (2) Data model [40]. With regards to the service interface layer, the STA defined a RESTful pattern, based on the OASIS OData standard, that allowed different STA services to exchange and filter entities defined by the STA data model. As for the data model aspect, the STA data model was based on the International Organization for Standardization (ISO) and *OGC* Observation and Measurement standard model [41]. As a result, the data model can interoperate and is backward compatible with the OGC Sensor Observation Service (SOS) Web service. The following UML diagram describes the entities of the STA data model. In the OGC STA, every Thing can have zero or more locations in space or time ((Figure 2). Furthermore, each Thing can have zero or more “Datastreams”. A Datastream is a collection of “Observation” entities grouped by the same “ObservedProperty”. An Observation is an event performed by a “Sensor”, that is a process producing a result with a value that estimates the ObservedProperty of a “FeatureofInterest”.

The OGC STA provided an interoperable framework with which to build the proposed IoCT. STA’s O&M-based data model and query functions have been shown to work for a very wide range of IoT systems from simple weather stations to complex drone systems. By using the OGC STA, we were able to develop an IoCT that interconnects heterogeneous IoT devices, data, and applications over the Web. In order to deal with the pandemic’s fast-changing requirements, IoT developers need an established working architecture that will work not only for today, but also for future, COVID-19 applications. In addition, healthcare applications are often near real-time and need to be scalable and performant, i.e., able to accommodate a very large number of devices that are sending high frequency data simultaneously without sacrificing performance. The goal for the IoCT is to build an interoperable foundation for future expansion and integration using various existing and new COVID-19 IoT applications.

### 2.3. Interior Space Modelling Using IndoorGML

Indoor spaces differ from outdoor spaces in many aspects. Basic concepts, data models, and standards of spatial information need to be redefined in order to meet the requirements of indoor spatial applications. The proper representation of indoor spaces is a key issue for indoor spatial information modelling and analytics. In recent years, the topic of 3D geospatial indoor modelling has been the focus of attention for location-based services and indoor navigation [42,43,44]. As the risk of COVID-19 virus transmission is higher in indoor environments, indoor space modelling is an important topic that facilitates the interoperability between different indoor and outdoor data collection methods and builds a consistent framework for collaborative research and development of the IoCT.

Aggregating different sensor observations for each room is essential for estimating the room’s COVID-19 risk and cleaning requirements. The visualization of Interior Space Risk State is another task which requires interior modelling. In order to represent the risk of infection and to identify which specific areas of a building require the most cleaning, the status of individual floors should be able to be viewed separately from other floors in the building. The risk of infection for various parts of each floor should be represented in a map with different ways of representing the data that is necessary for determining the risk in each area. Moreover, in order to visualize trajectories for contact tracing and to quickly identify the location of infection spreading behavior within an indoor space, the buildings should be visible as a 3D construct on the map. Therefore, in order to analyze the IoCT multi-sensors system and visualize it in indoor scenarios, an interoperable 3D building modelling standard, such as the “CityGML” Level of Detail 4 [45], “OGC IndoorGML” [46], building construction standards (e.g., “Building Information Modelling” (BIM), or “Industry Foundation Classes” (IFC) [47]), is necessary. The main concern for using those models is their fit and how often they are updated. Construction features of indoor spaces are not a major focus of COVID-19 workplace reopening scenarios. Instead, the aggregation of sensors in each room, and the connectivity between the rooms, is fundamental for risk assessment and tracing. Thus, the OGC IndoorGML is used for the IoCT indoor modelling. According to Ryoo et al. [43], the OGC IndoorGML can be used more effectively than CityGML or any other geometric representations of space for analyzing the trajectories of people inside buildings. This allows for more accurate appraisal of the types of intersection of trajectories, contact, and exposure for infection risk evaluation. Applications such as cleaning risk assessments for COVID-19 workplace reopenings that need to operate efficiently together with indoor scales, various sensors, and objects that are moving and changing over time would benefit from using the OGC IndoorGML. 3D geometry can be included in an IndoorGML document, and the overlap with other standards (e.g., OGC CityGML) can be addressed by adding external references. 

There were no specific standards in the field of indoor geospatial modelling until the OGC standard IndoorGML was introduced in 2014. The OGC IndoorGML intentionally focused on modelling indoor spaces using connected dual graphs for navigation purposes whilst considering various semantics [46]. OGC IndoorGML standard specifies an open data model and Extensible Markup Language (XML) schema for indoor spatial information [46]. Indoor space is comprised of connected constructs such as rooms, corridors, stairs, and elevators, all of which can be considered “Cells”. This sets it apart from other standards in the field of 3D modelling, such as CityGML or IFC, as they model the building features (e.g., walls, windows) instead of the indoor space itself. They also do not consider the connectivity and semantics of indoor spaces.

**Figure 3:**
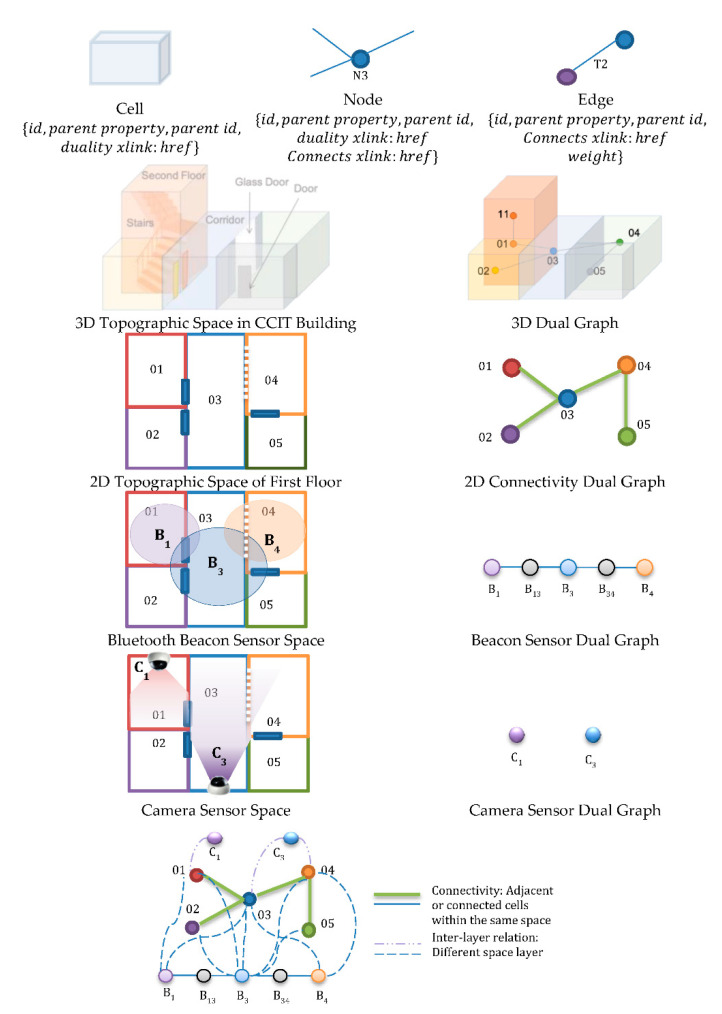
An Example of the *OGC IndoorGML* Data Model from the Basic Elements to Multi-Layered Space Dual Graph Model of the First Floor of the CCIT Building.

As shown in Figure 3, the nodes of the IndoorGML graph in this paper are considered the smallest organizational or structural units for the building and are called Cells [19]. Every Cell has an identifier (e.g., room number) and a location *(x*, *y*, *z)* to provide more precise location details. Cells are connected and have a common boundary with other cells but do not overlap with them. “Geometric” features and “Topological” relationships, such as adjacency and connectivity, amongst indoor cells can be defined by an IndoorGML graph [48]. The topological relationships in IndoorGML are explicitly described using the xlink concept of XML provided by Geography Markup Language (GML) and the referencing is realized with the use of href attributes (xlink:href). Semantics are also an important characteristic of the Cells in the IndoorGML. “Semantics” allow us to define cells which can be important for cleaning risk assessment. For example, the most commonly used areas are public rooms, corridors, and doors, and thus present higher risk. For this paper, an indoor space is represented as a topographic cellular space comprised of rooms, corridors, and stairs. At the same time, it is also represented as different cellular spaces with beacon or camera coverage Cells.

**Figure 4:**
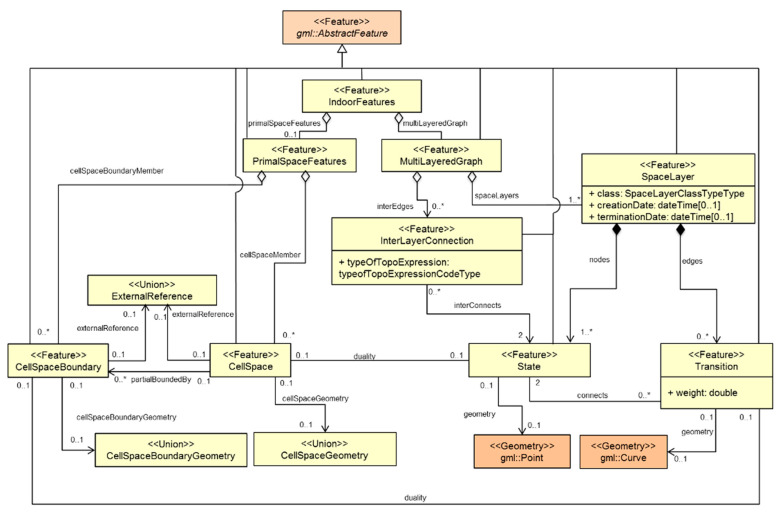
Multi-Layered Space Model Unified Modelling Language Diagram from [46]: in this figure, “*” denotes “many” instances in ‘0 to many” and “1 to many” relationship.

Each semantic interpretation layer creates a different indoor model, and each model forms a separate dual graph layer (e.g., connectivity, sensor) for the same cellular space. This multi-layered space model (Figure 3), is an aggregation of the space layers and inter-layer connections or relations. The Indoor GML for the implementation of the structure space model is shown in Figure 4. 

## 3. Proposed Interoperable IoCT System Architecture

**Figure 5:**
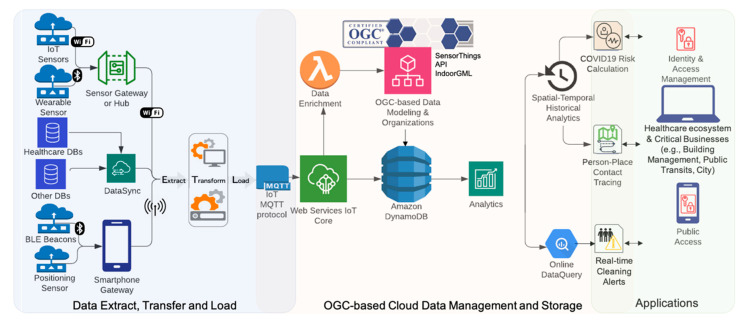
General Architecture for the Proposed IoCT Platform.

The following architecture was proposed for the IoCT in order to design, implement, and evaluate a scalable, interoperable design for incorporating various sensors, geospatial data infrastructures, and healthcare information for post COVID-19 reopening applications. Figure 5 shows the IoCT proposed architecture for interconnecting an Internet of heterogeneous COVID-19 system of systems with the interoperable geospatial IoT technologies using OGC standards. The following sections summarize this architecture in three parts: Sensor and Data Extract, Transfer and Load, *OGC*-Based Cloud Data Management, Storage, and Application layer.

The first section describes the “Extract, Transform, Load” (ETL) architecture for geospatial sensor data and resource datasets. Disparate geospatial and IoT data sources are available for monitoring and studying COVID-19 spread. The coordination of a diverse range of data requires a comprehensive communication, integration, and interoperability model. Existing IoT systems operate within silos of information, APIs, and proprietary data formats. Firstly, the proposed architecture aimed to aggregate heterogeneous and real-time COVID-19 data streams by extracting data from heterogeneous data sources. There were two types of location-based information used for the IoCT: Positioning and Proximity. GNSS-based positioning accurately (within two to five metres on average) estimates the outdoor location of a wearable device. Most proximity sensors only provide closeness information with a range of no more than five metres from a position that is usually represented by a Bluetooth beacon. Location information was integrated into a smartphone app in an edge gateway device for computation and the transference of data onto the cloud. The other data source for monitoring workplaces came from available data streams from smart camera and audio sensors. Smart cameras and audio sensors were attached to a Jetson Xavier NX development kit [49] which served as the edge computation device for deep learning (DL) computation and the IoT gateway. Various sensor data streams were transformed by data cleaning and preparation for contact tracing query and analytics. This vast amount of spatial-temporal data was then inserted into a data stream Management System (DSMS) in near real-time. After ETL, the sensor data loading modules streaming the disparate data sources into the cloud module can be developed using the OGC STA, an open geospatial IoT exchange standard. In the cloud data storage, these datasets need to be aggregated into a unified geospatial data model and encoding also known as the *OGC* IndoorGML hierarchy of indoor cell spaces.

The OGC-based cloud data management and storage section in Figure 5 presents a cloud-native OGC standard-based IoT platform for people and place data management. A cloud-native architecture (a container-based environment) was designed and developed to enable distribute, scalable, and flexible management and access of the IoCT datastores. Building a cloud-native architecture with open geospatial standards enables interoperability and scalability. The proposed cloud architecture was based on Amazon Web Services (AWS) and is capable of scaling out, and up, to handle the high-volume, high-velocity, single, or multiple, real-time data streams and user access. The proposed IoCT architecture is geographically scalable and considers spatial indexing technology. This scalable IoT data cloud architecture was designed in a way that was distributed, load balanced, and without a single point of failure. Kubernetes, a container orchestration framework, and AWS Managed Services were used as the building blocks. To get real-time insights into data streams and prepare them for analytics, we designed some enrichment functionalities using the Lambda function that included, location, semantics, metadata, collection method, or contextual information. To create an interoperable common operating picture for spatial data, we used OGC standards, data models, and encodings, in addition to the OGC STA to connect not only different IoT platforms, but also external geospatial applications and visualization tools. The OGC Standard-based data records published in the AWS IoT Core were stored in Amazon DynamoDB which functioned as a fully managed, No-SQL, scalable database. The proposed cloud-native platform is able to support a flexible security model thus allowing for a range of policies to be implemented. A security layer was also implemented in the cloud to support a centralized security model in order to integrate different design choices and cryptographic models as dictated by public health response. This integrated security layer worked with different systems and increased the system’s security with a cryptographic design which was not decoded for the cloud. Then, a publish/subscribe model for data delivery was developed, allowing for different levels of data access.

The last section discusses how two prototype applications were built based on the open geospatial architecture. Firstly, we demonstrated the interoperation of the Internet of disparate COVID-19 solutions and then contextualized them using open geospatial standards such as the OGC IndoorGML. Secondly, a new and unique geospatial algorithm was examined by building a person-to-place risk model for cleaning indoor spaces based on the colocation or co-movement patterns of people and places (e.g., a room) in an effective and interoperable way. For the visualization purpose of this research, the SensorUp Explorer developed by SensorUp Inc. was used and further developed as a spatiotemporal Web dashboard.

## 4. Experimental Design

This section discusses the experimental design related to a cleaning risk use case as the most important prevention activity in post COVID-19 workplace reopening with the use of the IoCT as a multi-sensor platform. To effectively integrate the multi-sensor system for cleaning risk analysis, a multi-criteria evaluation [50] was applied to identify, and rank COVID-19 risky behaviors based on an available multi-sensor system in the CCIT building at the University of Calgary campus. Since 80 percent of the data used by the proposed IoCT system was geospatially related, Spatial Multi-Criteria Decision Analysis (SMCDA) provided a superior framework for a variety of decision-making situations [51,52,53].

### 4.1. COVID-19 Risk Assessment Using IndoorGML

**Figure 6:**
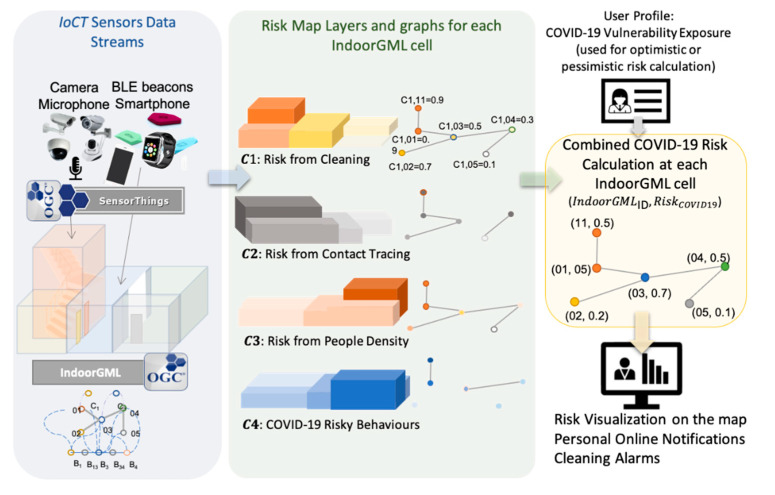
SMCDA *COVID-19* Risk Evaluation.

The SMCDA simultaneously represents decision spaces as well as criteria values based on attribute and geographic topology [50]. For this research, topological relationships from the OGC IndoorGML dual graph were used for risk aggregation for the multi-sensor system. A scientific SMCDA process can be put in place using the different steps shown Figure 6. In order to initialize the decision-making process for this paper, equal weights for various risk criteria map layers were considered. This helped ensure fast implementation and quick proof of concept.

The main step for risk criteria assessment was determining the factors affecting the risk of COVID-19 spread based on information from existing studies from both the World Health Organization (WHO) [27] and the Government of Canada website [28]. The number of active virus particles present in a place was considered the most important factor for determining the risk of infection [27]. Various transmission ways of SARS-CoV-2 transmission include airborne transmission caused by small droplets, and larger droplet transmission (droplets can survive up to several days on different surfaces) [54]. The term “viral load” will also be used to refer to the number of active virus particles present in a space. Virus particles live for different lengths of time, depending on a number of factors, the most significant one being surface material. Risk of infection for any particular IndoorGML cell space was modelled as the viral load within the space.

Assuming that a proportion of any average group of people is infected, the viral load within a space increases along with the number of people occupying it, the amount of time the people spend in the space, and the actions of the people within the space. Talking loudly, exercising, and coughing expel more droplets into the environment than other activities, and thus increase the viral load within the space. The virus also passes from surface to surface through touch, so touching surfaces without cleaning hands in between also increases the viral load within the space. The viral load was broken down into a hierarchy of smaller cause similar to a root cause analysis in order to evaluate the different factors. The following layers represent the respective criterion maps. Effective parameters were identified based on available sensors and data according to the implemented IoCT multi-sensor system. The viral load risk criteria are listed as follow:

- C1**:** Risk from Cleaning: Cleaning schedule reported on a smartphone app based on the time that had elapsed from the previous cleaning. For this paper, the cleaning frequency for each room was every 6 h, meaning that after six hours the risk is maximized at one whereas immediately after cleaning it is at 0. C1 is a spatiotemporal map layer comprised of OGC IndoorGML cells with values between 0 and 1.
- C2**:** Risk from Contact Tracing: Proximity tracing map extracted from beacons which includes a trajectory map of traced people on an OGC IndoorGML graph. These trajectories show the location of the cleaner and the number of people in a place. If a person identifies himself or herself as a COVID-19 infected person, the historical trajectories can be used for the contact tracing map layer calculation.
- C3**:** Risk from People Density: Gathering restriction map from smart cameras which includes the number of people over each IndoorGML node. This value changes over a range of 0–1 based on the number of people divided by the capacity of the room (which can be assigned or generated from the area property of an IndoorGML cell node). This information is reported online and aggregated once the room is cleaned.
- C4**:** COVID-19 Risky Behaviors: Risky behavior violation map which includes the number of incidents or violations (number of people violating social distancing, hugging, people touching common surfaces and objects, talking loudly, exercising, coughing, and sneezing). This value is a weighted average of the risky behavior factors (number of violations) over the frequency of cleaning (normally six hours for each room). This layer was generated using smart cameras and audio sensors based on the number of detected risky events using deep learning algorithms as described in the following sections.

As we progress with COVID, various criteria have been introduced and evaluated in COVID-19 spread risk [54,55]. Transmission ways of coronavirus and prioritizing their importance are still under debate and more studies are underway to understand transmission ways better [56]. Although the risk calculation could be very complicated based on time, room volume, air circulation, etc. [57,58], our research’s scope includes a general risk assessment function which is a simple weighted average. The *COVID-19* viral load risk function was modelled using a weighted average of risky criteria (as mentioned above) over each IndoorGML node (e.g., a meeting room). For the initial prototype calculation of risk, Equation (1) was used:

(1) $$\{\begin{array}{c} \;Risk_{COVID19}\;=\;W_{1}C_{1}+W_{2}C_{2}+W_{3}C_{3}+W_{4}C_{4}\\ W_{1}=W_{2}=W_{3}=W_{4}=1,\;to\;simplify\;the\;model \end{array}$$

For simplicity sake, we assigned each of the above map layers with a similar weight and computed the risk factor over each *IndoorGML* node using an aggregation function.

However, this risk function can be easily manipulated and configured by the users on the client side. So, we evaluated a set of different weights and evaluated them in Section 5.7. In this new risk model, the wights are as follows:

- Risk from Cleaning $:\;W_{1}=0.1$*:* since cleaning is a measure of potentially unknown risks of COVID-19 transmission, such as airborne particles passing through the ventilation.
- Risk from Contact Tracing: $W_{2}=0.4$*:* it is one of the strongest risks for the place
- Risk from People Density: $W_{3}=0.3$*:* people using the space or engaging in risky behaviors in the space are stronger indicators and higher weight.
- Risky behaviours:$\;W_{4}=0.2$*:* We assumed the number of people being the strongest due to the prevalence of airborne virus being multiplicative on the number of people in the room, whereas risky behaviors are less frequent, and therefore harder to weight ($W_{3}=0.2$).

Moreover, the above Equation (1) does not take into account the duration of time spent occupying the space, the actions taken, and the decay of the virus particles over time. The algorithm restarts the people count and cough numbers after the space is cleaned. A slightly more complicated set of equations (Equation (2)) expands on the simple risk calculation by taking into account the amount of time that people remain in a particular space, and the decay of the virus particles over time (assuming the worst-case scenario of 72 h for all of the particles deposited to become inactive).

(2) $$\{\begin{array}{c} C_{3}=\;\mathrm{number}\_\mathrm{of}\_\mathrm{people}\;\times \;\mathrm{person}\_\mathrm{dwell}\_\mathrm{time}\left(\mathrm{hours}\right)\times \frac{72-\;\mathrm{time}\_\mathrm{since}\_\mathrm{departure}\left(\mathrm{hour}\right)}{72}\;\\ C_{4}=Average_{\mathrm{LastRiskyBehavior}}\times \frac{72-{\mathrm{Time}}_{\mathrm{LastRiskyBehavior}}\left(hour\right)}{72} \end{array}$$

For future research, we will consider different risk profiles (i.e., optimistic and pessimistic) for various user groups (e.g., pessimistic risk profile for COVID-19 vulnerable people).

### 4.2. Interoperable IoCT Using STA

**Figure 7:**
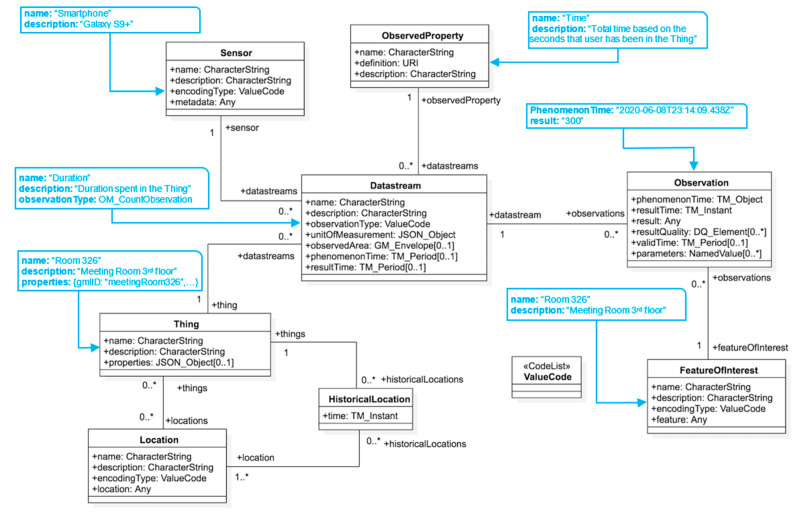
An Example of Modelling *OGC STA* for a Selected *IndoorGML* Cell in this figure, “*” denotes “many” instances in ‘0 to many” and “1 to many” relationship.

In order to integrate multiple COVID-19 sensor systems, the OGC STA was used to support the interoperability between the sensing layer, cloud data management, and cleaning risk assessment application. Figure 7 shows an example of the OGC STA data model being used in the cleaning scenario for a specific Thing—an IndoorGML cell.

Every IndoorGML cell has a Location in space and time. This geospatial encoding was performed by GeoJSON (Geographical JavaScript Object Notation) [59]. Every sensor was referenced by the IndoorGML cell in which the sensor was installed. Each Thing can have multiple Datastreams, which are collections of Observation entities grouped together using the same Observed Property. For the cleaning use case, a different Datastream for each sensor’s phenomenon was used. Each Datastream contained a Sensor and an ObservedProperty. This refers to the instruments that can observe a phenomenon. For this paper, eight different Datastreams were defined, including, proximity, density, and coughs. An ObservedProperty specifies the phenomenon and also contains the unit of measurement. A Datastream can have several Observations, and they dictate the value for the phenomena encoded by the OGC Observations and Measurements (OM). For our example, this can refer to the values taken from a sensor measurement. FeatureOfInterest identifies the characteristics of the Thing. The Thing entity is an IndoorGML cell and the FeatureOfInterest entity describes the characteristics of this cell. For example, Figure 7 shows that “Duration” spent by a smartphone user in a room recorded within the proximity of a BLE beacon is considered a Datastream entity that kept the “Time” duration in seconds as an ObservedProperty. This Datastream entity used “Smartphones” as the sensor entity to keep Observations which are the duration of time that users spend in each cell in seconds.

Table 2 lists all Datastream entities that were used together with their sensing profile, whereby each property indicates the type of format that was encoded. For this research, all of the observations were sent to the Amazon IoT Core using smartphones and the Jetson Xavier NX development kit [49]. The next step was to map observations to an instance of the *OGC STA* endpoint using the Amazon Lambda functions. Interested readers can see and test the JSON payloads that were used to send all eight types of observations in Supplementary Materials.

## 5. Results and Discussions

### 5.1. Smartphone Cleaning App

**Figure 8:**
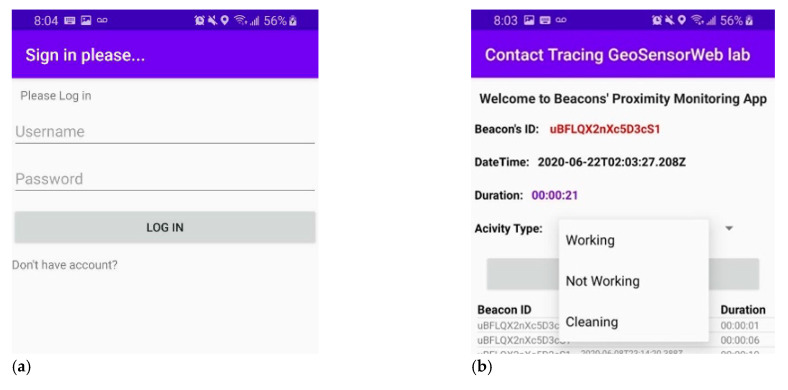

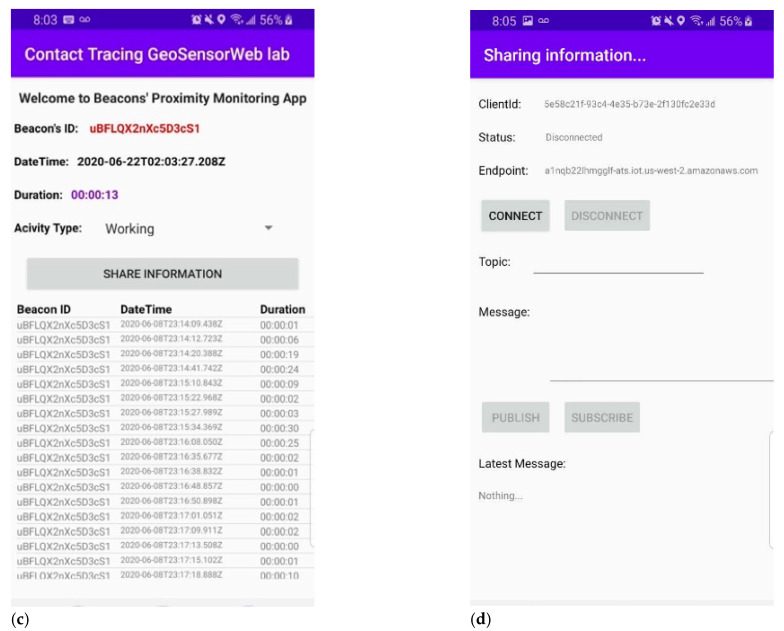
The developed BLE contact tracing mobile application. (**a**) Sign in/up process for using Amazon Authentication Service using the Amazon Cognito Product; (**b**) Showing three different types of activities; (**c**) Collecting Observations including Date, Time, BeaconID, and Time Duration, and storing them in the internal SQLite DB; (**d**) Publishing internal information to Amazon Cloud using MQTT Protocol.

Cleaning activities play an important role in reducing the risk of being exposed to COVID-19. Three types of user activities were defined for the purposes of this research: Working (i.e., the user is busy working), not working (i.e., the user is either a visitor or having time off), and cleaning (i.e., the user is a staff member who is either cleaning or disinfecting the room). As seen in Figure 8a, these three different activities were taken into consideration by the mobile application and the user-selected types of activities were internally stored in their mobile phones.

We assumed that after the cleaning activity was carried out, the risk of any COVID-19 viral load being present returned to zero. Over time, interactions between users and the space such as coughing, talking, and touching surfaces would again increase each room’s risk (Equation (2)). If a cleaner specifies in the mobile app that cleaning is done, the room will be marked as “cleaned”, and the risk will go down to zero. Cleaning staff, based on the COVID-19 dissecting rules and regulations forced by the facilities, are trained and clean the room using advanced cleaning equipment (e.g., electrostatic sprayers), which kills 99% viruses. This cleaning activity ensures the virus is killed, and there is no chance for cross-contamination. It is reasonable to assume that the facilities will take precautions with cleaning as much as possible. However, if this assumption is not valid, the risk will be increased over time, which complicates the calculations and increases virus spread and true-positive alarms. Considering cleaning activities resets the risk calculations for the final risk map and reduces false-positive COVID-19 notification alerts. In the future, we are going to evaluate standard-level cleaning activities for COVID-19 using smart cameras automatically. Furthermore, cleaning should include enhanced space ventilation, as airborne particles are remarkably decreased by adequate ventilation.

For this research, a virus transmission interval is assumed to be a time interval of 15 min. In other words, if user A was interacting with a room that had been used by a positive COVID-19 infected person, user B, the system would notify user A of probable exposure to the virus. If we consider the situation in which cleaning activity took place after user B left the room, the risk of being exposed by the infected place would be zero. This case can be considered a false positive notification alert for user A. As a result, the proposed system can considerably reduce false positive notifications by using different types of activities. A demo scenario of cleaning person is presented in Supplementary Materials and the trajectories of both building cleaners and visitors is shown in Supplementary Materials.

### 5.2. Proximity-Based Contact Tracing

For the purposes of this research the third floor of the CCIT building was selected for an experiment. After extracting the related metadata such as room names for the rooms from the IndoorGML, 12 Estimote Proximity beacons were spatially distributed between 12 different cell spaces. The contact tracing technique applied for this research was designed in a way that protects user privacy. The application detects the proximal appearance of users within the proximity zone of each beacon by considering the value of the Received Signal Strength Indicator (RSSI) that was broadcasted by the beacons. The duration of appearance of the user in the proximity zone defined for each beacon and the corresponding date and time information for this proximal appearance are the only information stored in the internal storage of mobile phones. Figure 8b shows a screenshot of the developed mobile application for collecting different types of observations including BeaconID, time, date, and the duration that the target user spent in the proximal zone of each beacon. Assuming that the incubation period of COVID-19 is two weeks, the application will work as a background service that saves data internally for a two-week period.

**Figure 9:**
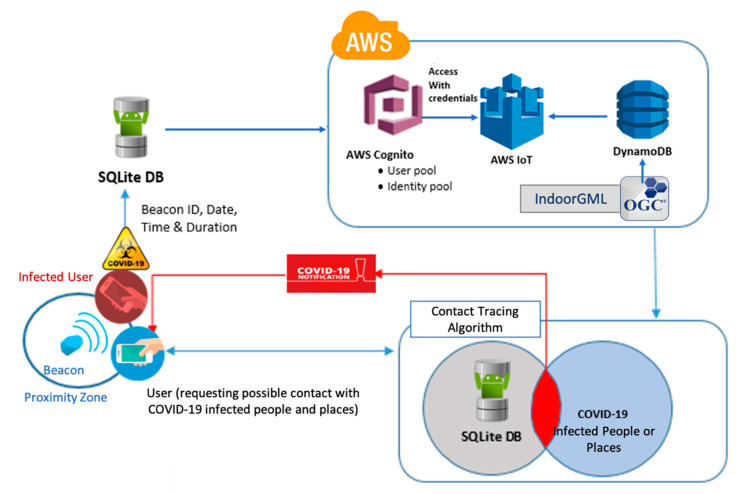
The Architecture of the Beacon-Based Contact Tracing Mobile Application Using Amazon Web Services.

In situations in which the user becomes a positive COVID-19 case, he/she can voluntarily share data captured within the past two weeks with the backend database management system. An AWS product Amazon Cognito was used to control user authentication and access to data storage. As shown in Figure 8c, users are required to sign in/up for an Amazon Cognito account in order to share their information. After signing in as an authorized client, users can publish their internal information to the Amazon cloud as shown in Figure 8d. All of the data related to the COVID-19 cases will be stored and managed in the DynamoDB database in the Amazon cloud. Our developed application was connected to the DynamoDB using another AWS product, the IoT Core. When new data is added to cloud storage, the contact tracing application will look for any matches between the backend data and the data stored internally in the user device. If it finds any matches that show that a confirmed COVID-19 positive case and the target user were close to each other for more than 15 min, the application will then notify the target user about potential exposure to COVID-19 and alert cleaning staff to disinfect the place. This process is shown in Figure 9. A demo of people trajectories is shown in Supplementary Materials.

There are various methods for indoor positioning, such as WiFi, BLE beacons, or dead reckoning. Using BLE technology is cost-effective compared to other indoor positioning techniques, which use maintenance, installation, and cabling costs. Generally, Bluetooth devices cost ~20× less than WiFi devices and have a similar WiFi accuracy [60].

In this paper, we focused on BLE proximity detection for contact tracing instead of precise positioning. Three categories of user location will be of importance for this paper including immediate (less than 60 cm), near (1–6 m), and far (>10 m) distance of the Bluetooth receiver from active BLE beacon. On the other hand, it was still a challenge working with BLE signals that are interfered with by structures. Indoor setting and layout have direct effects on radio waves used in Bluetooth technology. Another challenge was that the different beacon types and battery states produce different signal strengths, so using one beacon library for all types of beacons was problematic. 

In this paper, an active BLE beacon is placed in each IndoorGML cell (e.g., room). Moreover, we focus on proximity detection (i.e., immediate (within 0.6 m away), near (within about 1–8 m), and far (is beyond 10 m) distances from the active BLE beacon) to make indoor spatiotemporal trajectories using IndoorGML cell connectivity. We avoided having to determine the exact range by way of careful beacon placement to prevent overlaps. In the context of COVID-19 spread, locating in the immediate and near distance from the infected host would be dangerous for coronavirus transmission (through droplet transmission). Accordingly, different health organizations such as WHO recommended two meters distance from others. As a result, proximity detection should be of more importance in the COVID-19 context. In other words, considering precise positioning would only increase the computation cost in this specific application. Describing an indoor location using IndoorGML graph cell also helps with privacy. Considering privacy concerns for individual tracking, especially in indoor environments, we believe that proximity positioning respects user privacy more than precise positioning. 

Depending on the size of the data, type of beacons, and network bandwidth, mobile proximity detection performance may differ. In our experiment, various beacons such as Estimote (https://estimote.com/), Accent Systems (https://accent-systems.com/) and Radius Networks (https://www.radiusnetworks.com/) have been evaluated using the developed app on the Samsung Galaxy S9 smartphone. Our results demonstrated that the app could capture a beacon’s proximity of fewer than 60 milliseconds, which is enough for our case study. The complexity of the position determination depends on the beacon software development kit; however, the complexity is O(n) in the worst-case scenario. Concerning the duration spent in a room, we detected and recorded durations of less than five seconds when walking past beacons in a corridor. Significance of time for the sake of COVID-19 risk was not considered important for durations less than 15 min, which was standard practice. So, our sampling and recording intervals were much better than was required for COVID-19 risk evaluation.

The mobile application publishes a JSON payload to the AWS IoT Core cloud data management system in which:

- Online service: A single record showing the presence of a user in the proximity of an active BLE beacon is published to the AWS IoT core.
- Offline service: An array of records showing the user’s pretenses in a time window is published to the AWS cloud.

A JSON payload showing a single enriched proximity location captured by the developed smartphone app is shown in Supplementary Materials. For more information regarding contact tracing app can be found in [61].

### 5.3. Video-Based People Density

This section discusses the experimental design for our camera surveillance for counting people, People Density, or the number of people who entered or left a geofence polygon area. For indoor spaces, Physical Distancing rules result in restrictions on the number of people occupying a space. The input for the DL models was online video feeds of fixed cameras focused on the regions of interest defined as IndoorGML cells (e.g., rooms, corridors, lobbies, elevators, stairs, and coffee places). Some cameras might even be able to cover multiple regions of interest (IndoorGML cells), depending on where they are installed and if the spaces are separated by glass walls or windows. An alarm can be triggered by the number of people entering or exiting a region (identified in the camera image) if the density of people exceeds the density of that area. Moreover, the number of people violating physical distancing rules can be identified and reported to the IoCT.

**Figure 10:**
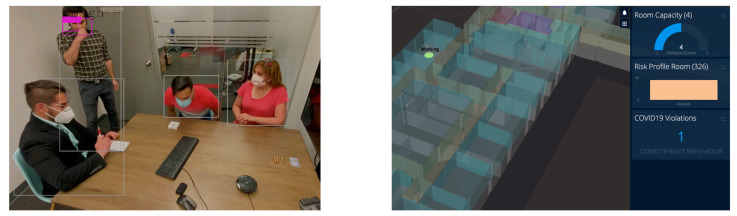
A Meeting Room Camera Frame Shows Detected People (**Left**) and Gathering Restriction Alarm on the Dashboard (**Right**).

For our cleaning use case demo (Supplementary Materials), we considered a meeting room as an IndoorGML node (Room 326) with a four-person capacity. For this demo, the OGC indoorGML was used as it offered the following advantages: IndoorGML cells were defined as the geofence; the geometry and area of each cell (geofence) were calculated and the location of each indoorGML cell (the centroid of the geofence) was used for the enrichment of the camera data. The number of people entering or exiting each cell was monitored. People in each frame were detected in real-time using a pre-trained You Only Look Once (YOLO) model [62] and the results were then published as an MQTT message to the AWS IoT Core. On the backend, the maximum allowed people in a cell, or cell capacity, was either assigned by the building management, or calculated by dividing the cell area into squares of six feet two inches. The “Gathering Restriction”—the number of people over each IndoorGML node—was then calculated. This value changes over a range of 0–1 based on the number of people divided by the capacity of the room. Should the number of people exceed the cell capacity, a Gathering Restriction alarm would be generated for the cell. The following figure (Figure 10) shows a frame of the meeting room, detected people, and Gathering Restriction alarm. The video demo of this scene is attached in Supplementary Materials which shows the people count online when they enter or exit the room.

### 5.4. Video-Based Physical Distancing

**Figure 11:**
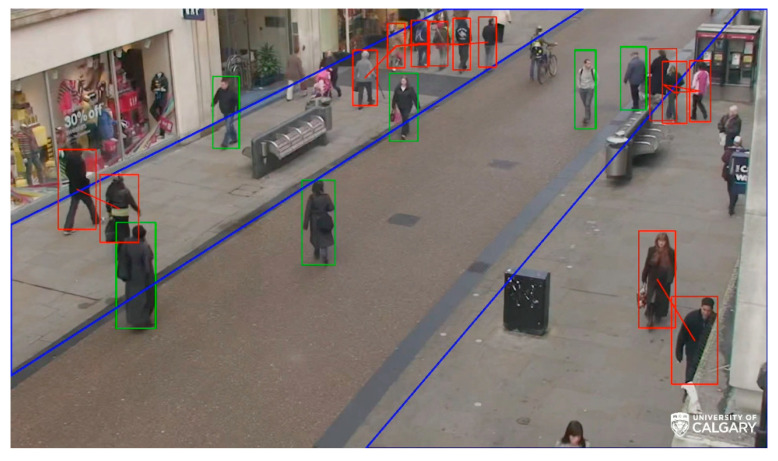
A camera frame showing detected people and Physical Distancing violations (red bounding boxes show approximate physical distances of less than two metres; green boxes show allowed physical distances; and blue lines indicate geofences area).

Physical Distancing was monitored for each cell using a pre-trained YOLO model for detecting people in that cell. Relative distance was then calculated as follows: The pairwise distance between two people is the distance between the two similar corners of their bounding box. In order to minimize the camera’s vanishing point effect, the distance was then compared to their bounding box diameters. If the distance was less than the longest diameter, it was assumed that the relative distance between those people was violating the Physical Distancing rule. For the following example, the view from a fixed camera was divided into several polygons (geofences). This can result in the creation of separate geofences (indicated by the IndoorGML nodes if they were in the building) from the camera’s viewpoint. The number of people per geofence polygon and the number of times that people were closer than two metres were reported to the IoCT. The following figure (Figure 11) shows a frame of multiple geofences in an outdoor area, the detected people, and the Physical Distancing violations. The video demo of this scene is attached in Supplementary Materials which shows the people count online when they entered or exited the geofences, as well as the physical distancing violations. Outdoor geofences can be connected to the IndoorGML graph nodes.

### 5.5. Video-Based Risky Behavior Detection

**Figure 12:**
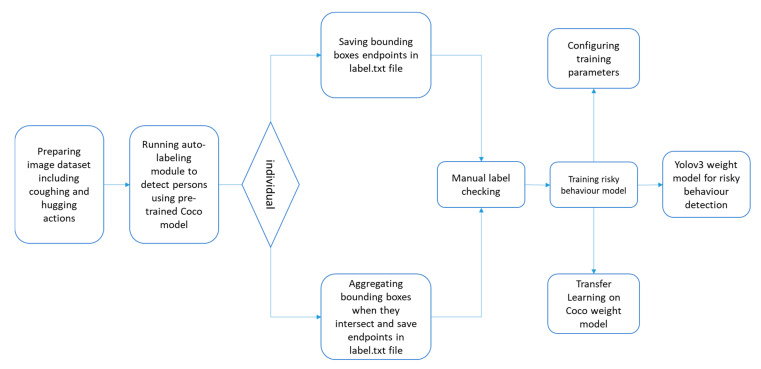
Behavior Detection Training Flowchart.

Camera stream processing is a popular and quick way to detect objects. Human behaviors and actions can be detected as objects from the video frames using a trained deep learning model. For the detection of risky behaviors such as coughing, hugging, handshaking, and doorknob touching, the You Only Look Once version3 (YOLOv3) which is suitable for real-time behavior detection for online video streams, was trained and applied [63,64]. This library classifies and localizes detected objects in one step with a speed of faster than 40 frames per second (FPS). We considered two main types of risky behaviors for COVID-19 indoor transmission: Group risky behaviors (e.g., hugging) and individual risky behaviors (e.g., coughing). Figure 12 illustrates how to train a model for COVID-19 transmission risky behavior detection using YOLOv3.

In total, 603 images for coughing, 634 images for hugging, 608 images for handshaking, and 623 images for door touching were used from COCO dataset [62] for transfer learning for the pre-trained model (YOLOv3). These images were taken from free sources found through Google image searches. For labelling objects, a semi-automatic method was applied. Darknet library was also used for training. For individual behaviors, all of the people in images were detected and labelled in a text file whilst the algorithm aggregated intersected bounding boxes of people into a single bounding box. As wrong labels might be generated, the images should be manually checked to correct misclassified objects. For this step 80 percent of the images were selected for training and 20 percent for testing. To increase the accuracy of this model, the configuration in Table 3 was used.

To increase training accuracy and speed, a transfer learning process was applied. The base layer is a pre-trained YOLOv3 that uses the COCO dataset for all of the layers of our model except the last. Transfer learning helps with training by exploiting the knowledge of a pre-trained supervised model to address the problems of small training datasets for COVID-19 risky behaviors [65]. To evaluate the accuracy of the model, we tried to check the results for different video datasets by exporting all of the frames for detection under various circumstances for the metrics listed in Table 4.

After studying the outcomes, we found that the “hugging” and “handshaking” classes experienced the highest false negative results compared to coughing as the larger dataset was being prepared for training. It appeared that hugging and handshaking (grouping actions) were more varied in terms of the types of handshaking and hugging. Therefore, training precision could be improved with the preparation of more varied data. Moreover, some of the false positive results for coughing showed that in most cases, moving a hand near the face was detected as coughing, regardless whether it had actually taken place. Furthermore, the number of false negatives increased in a more populated area. Detected touching behavior results demonstrated high numbers of false negative cases. About 75 percent of false negative cases occurred when the predictor incorrectly detected small objects. Therefore, specifying limitations for box sizes and level of confidence for the predictor can reduce false negatives. The results of evaluating precision, recall, F-score, and number of samples for each behavior action class is listed in Table 5.

### 5.6. Audio-Based Risky Behavior Detection

**Figure 13:**
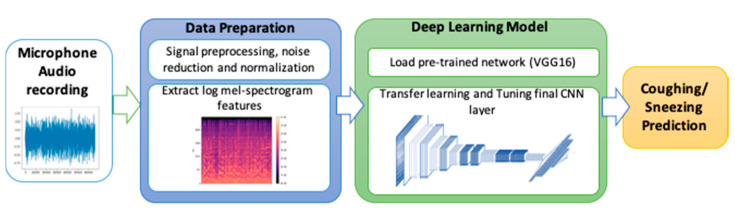
Methodology of the Audio Deep Learning Cough Detection.

This section examines an audio classification algorithm that recognizes coughing and sneezing using an audio sensor with an embedded DL engine. The methodology for audio detection is shown in Figure 13. This figure shows the four main steps of the audio DL process.The recording needs to first be preprocessed for noise before being used for extracting sound features. The most commonly known time-frequency feature is the short-time Fourier transform [67], Mel spectrogram [68], and wavelet spectrogram [69]. The Mel spectrogram was based on a nonlinear frequency scale motivated by human auditory perception and provides a more compact spectral representation of sounds when compared to the STFT [3]. To compute a Mel spectrogram, we first convert the sample audio files into time series. Next, its magnitude spectrogram is computed, and then mapped onto the Mel scale with power 2. The end result would be a Mel spectrogram [70]. The last step in preprocessing would be to convert Mel spectrograms into log Mel spectrograms. Then the image results would be introduced as an input to the deep learning modelling process.

Convolutional neural network (CNN) architectures use multiple blocks of successive convolution and pooling operations for feature learning and down sampling along the time and feature dimensions, respectively [71]. The VGG16 is a pre-trained CNN [72] used as a base model for transfer learning (Table 6) [73]. VGG16 is a famous CNN architecture that uses multiple stacks of small kernel filters (3 by 3) instead of the shallow architecture of two or three layers with large kernel filters [74]. Using multiple stacks of small kernel filters increases the network’s depth, which results in improving complex feature learning while decreasing computation costs. VGG16 architecture includes 16 convolutional and three fully connected layers. Audio-based risky behavior detection is based on complex features and distinguishable behaviors (e.g., coughing, sneezing, background noise), which requires a deeper CNN model than shallow architecture (i.e., two or three-layer architecture) offers [75]. VGG16 has been adopted for audio event detection and demonstrated significant literature results [71]. The feature maps were flattened to obtain the fully connected layer after the last convolutional layer. For most CNN-based architectures, only the last convolutional layer activations are connected to the final classification layer [76].

The ESC-50 [77] and AudioSet [78] datasets were used to extract cough and sneezing training samples. The ESC-50 dataset is a labelled collection of 2000 environmental audio recordings suitable for benchmarking methods of environmental sound classification. AudioSet consists of an expanding ontology of 632 audio event classes and a collection of 2,084,320 human-labelled, 10 s sound clips taken from YouTube videos. Over 5000 samples were extracted for the transfer learning CNN model which was then divided to train and test datasets. We examined the performance of the trained CNN models using coughing and sneezing. The results are shown in Table 7.

### 5.7. Risk Calculation and Visualization

**Figure 14:**
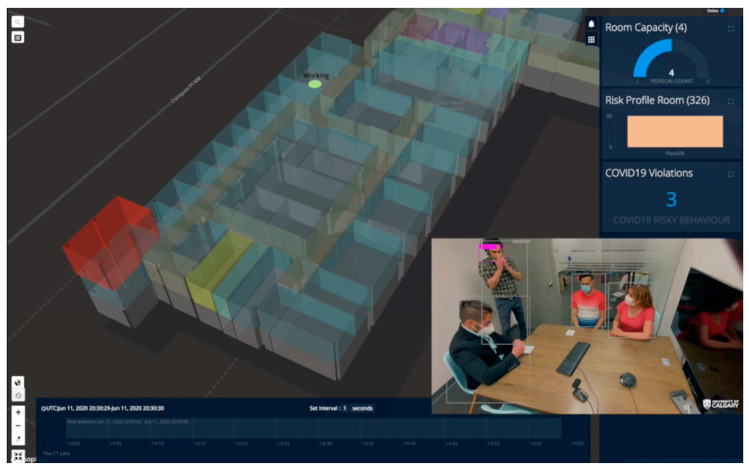
A Sample Screen Shot of Risk Calculation and Visualization when Four People are in Room 326 and a Cough is Detected (the Full Video Demo is Available in Supplementary Materials).

To demonstrate risk calculation using Equation (2), we evaluated the proposed IoCT using the following cleaning use case scenarios. In meeting room number 326 of the CCIT building, the number of people increased as people entered the room, and this event was detected by a smart camera in the room. The number of people was shown online in the video frame and map visualization browser in green until the room capacity (five) was reached. When the fourth person came in (room capacity is assumed to be three), the alarm notification for “Room exceeded capacity” is shown. After that, a person coughed in the meeting room, and this event was detected by both the smart camera and audio sensors. A notification showed “Cough detected”. Then, the person who coughed opened the door and this event was detected by the smart camera. A “High-risk behavior detected” notification was shown. The risk profile at that moment exceeded the threshold of 0.7 and a notification was sent to the people in room, and to a cleaner. The color of the room polygon turned red indicating high risk and the room polygon was extruded (i.e., the polygon height increases) proportional to the risk value. People started to leave the room causing the risk from People Density to go down, but the risk is higher than at the very beginning as a coughing event had occurred. The total risk value of the meeting room falls but remains higher than before the risky behavior (i.e., cough) took place. The cleaner closer to the room changes his activity status to cleaning (shown by an icon on the map) and moves closer towards the room (from elevator to room). The cleaner trajectory alongside the other people trajectories extracted from BLE beacons were visualized too. After the cleaning activity, the room’s total risk level goes back down to zero and the color of the room polygon changes back to green. The video demo of this scene is attached in the Supplementary Materials which shows the risk profile of the room. A sample screen shot of the Supplementary Materials demo video is presented in Figure 14.

**Figure 15:**
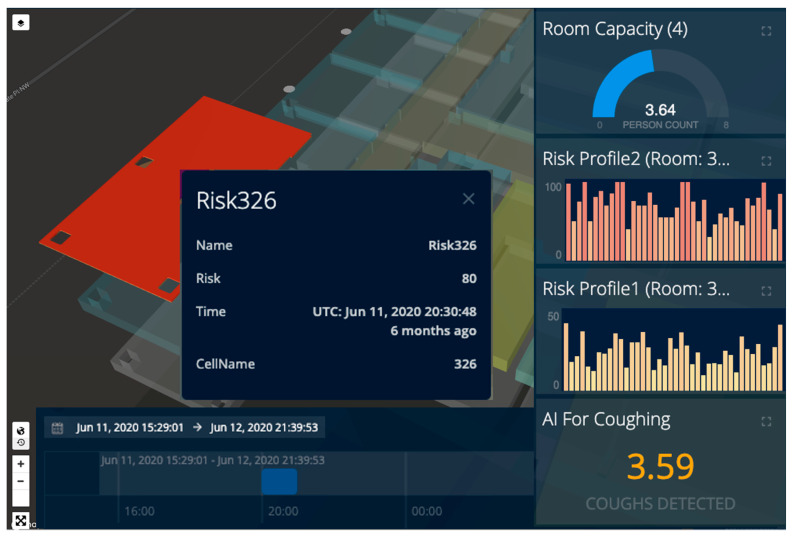
Risk profiles for room 326 over the period of 40 min from 20:00 to 20: 40 p.m. on 11 June 2020: Two gadget shows risk profiles and each bar demonstrate calculated risk for every minute.

To evaluate the impact of various weights assigned to different map layers, we used two sets of weights for map layer aggregations on the client side: $Profile\;1:\;W_{1}=W_{2}=W_{3}=W_{4}=1$; and $Profile\;2:\;W_{1}=0.1$, $W_{2}=0.4$, $W_{3}=0.3,$ and $W_{4}=0.2$ as mentioned in Section 4.1. Figure 15 shows two risk profiles for room 326 over 40 min from 20:00 to 20: 40 p.m. on 11 June 2020.

Evaluating precision, recall, and F-Score of video-Based and audio-Based risky behavior detection are listed in in Table 5 and Table 7 accordingly. Table 8 includes time performance of different developed functionalities (e.g., video-based person density, video-based physical distancing, video-based risky behavior detection, and audio-based risky behavior detection) on various platforms such as Jetson NX, laptop, and android smartphone. The performance of using a deep learning engine is highly dependent on Graphics and Computing processors. Therefore, the performance of those functionalities is evaluated on a laptop with more robust processing units. The laptop has NVIDIA GeForce RTX 2070 with 7.5 computation capabilities and a Core i7. Therefore, the performance on Jetson NX is lower than on the laptop. The best performance values are video-based risky behavior detection because they only involve the object detection task. Audio-based risky behavior detection segments the voice in specific time frames and converts them into spectrogram images. Voice patterns are detected in images using the VGG model. Therefore, the time of processing for audio is higher than video object detection. Video-based people density and video-based physical distancing give worse performance values than simple object detection regarding complexities in tracking functions.

## 6. Conclusions

This paper presents an Internet of COVID-19 Things platform called IoCT which offers two main contributions: (1) The design and development of a low-cost, real-time, comprehensive situational awareness for workplace reopenings after COVID-19; and (2) Interoperability through the open geospatial standards for indoor COVID-19 person-to-place risk assessment. In addition, the proposed platform is able to be applied to any kind of sensor and for use with different applications.

The proposed IoCT platform offers an easy connection between software and hardware which is necessary to achieve a global-level COVID-19 pandemic situational awareness. At the software level, a cloud architecture was developed for the IoCT, and the Sensors incorporated in this study are able to be included into it with minimal effort. At the hardware level, it offers a plug and play connection which will be explored for future research. It offers the possibility for scaling and access to a large number of low-cost sensors (manufactured by different companies) with an interoperable IoT design using the OGC STA as a conceptual modelling layer on top of the AWS. Furthermore, it provides the option of expansion because of the many compatible components which lead to the schematics being fully available.

In order to validate the proposed architecture, the IoCT sensor network was created and validated using multiple Things, Sensors, and Datastreams. Using the case of a scalable and connected COVID-19 IoT system, we deployed an interoperable sensorized platform to create a comprehensive picture for a post COVID-19 workplace reopening. A cleaning use case was developed for the University of Calgary campus to validate this. This platform was developed using an Android smartphone and Jetson NX, and applied the use of various sensors including BLE, camera, and microphone to provide many benefits. A network with 2 IoCT Things and 20 Sensors was successfully deployed. Each IoCT Thing was designed based on the IoT paradigm and can be considered a smart object that is permanently connected using the Internet Protocol.

Another benefit of using open standards is that they offer interoperable applications that facilitate access to data and reusability. A Web client was deployed to consume data for the OGC STA provided by the IoCT platform. The OGC STA offers easy and agile access to sensor data using IoT paradigms. Moreover, the OGC IndoorGML allows for the aggregation of various cameras and contact tracing systems that can work together in a common indoor risk model and exchange various data within the space model for risk calculations. The OGC IndoorGML model can be used for various trajectory mining as well. The IoCT can be used for person-to-place interactions in order to identify those who may have been in close contact with an infected person, or with a virus-contaminated place. Moreover, the proposed system will inform people to take appropriate actions such as cleaning, social distancing, testing, isolation, or choosing safe pathways and locations. This paper improves both the quality and speed of pandemic emergency response by enabling IoT system interoperability and unlocking necessary information for real-time decision making, as well as accelerating new application development that is interoperable, scalable, and extensible.

Our future work will explore the interoperability between various BLE systems and standards to achieve plug and play contact tracing apps with various contextual information [79]. Another area for future research would be applying different data analysis to the indoor trajectory data provided by the IoCT platform [80]. For that we would attempt to obtain different metrices for person-to-place scenarios using an aggregation of the camera and BLE sensors for trajectory estimation [61]. This analysis will include spatial-temporal methodologies for real-time event detection using the deep learning module.

## Figures and Tables

**Table 1:** Cleaning Use Case Definition as an Example of *Person-to-Place* Scenario.

Personas	Sarah (cleaner) wants to know:
	If an office was used; If a sick person was present; If the people density in each office is regulated; If people are following social distancing rules; If the cleaning risk for some rooms is high, she will receive the appropriate alerts to close and disinfect the actionable list of contaminated places to prevent further transmission.
Multi-Sensors System	A combination of the following sensors:
	Proximity sensors (i.e., beacons) for the following observations: ⚬ Relative closeness, or distance between a person and a beacon; ⚬ Indoor trajectories; ⚬ Density: Number of people in proximity with a beacon.
	GNSS location for outdoor trajectories.
	Cameras (security) for the following observations: ⚬ Density: Number of people tracked by a camera; ⚬ Social Distancing: Camera proximity app will notify if people are less than 2 m apart; ⚬ Coughing Behavior: Number of coughs, open coughs, hand coughs, and arm coughs result in different levels of contamination; ⚬ Touching Behavior: Cameras can detect if people touch the contaminated surfaces, doorknobs, or their faces; ⚬ Potential Detection: Automatic detection of people’s activities, for example, cleaning activities being carried out in a room, or wearing a face mask.
	Audio Sensors: ⚬ Coughing Behavior: Number and type of cough, dry cough versus wet cough, open coughs (without covering with hand/arm), arm-covered coughs, and hand-covered coughs result in different levels of contamination; ⚬ Density: Number of people based on human voice detection and noise levels.
	Other Potential Sensors: Thermal cameras for detection of fever (one of the most common symptoms of *COVID-19*). For example, infrared thermal camera scanning can be used at entrances to recognize any persons (including visitors) with fever at the first point of entry. Such sensors can be connected to the IoCT by publishing messages to the cloud based on the *OGC* standards described in this paper.
Related Use Cases	Alarms and Notification: ⚬ Self-Isolation; ⚬ Risk for a user group at work; ⚬ A *COVID-19* sick person present in that space; ⚬ Safe places and less crowded paths for *COVID-19* vulnerable user groups.
	Workplace Eligibility: ⚬ The manager of a business wants to know whether any employees have been in contact with an infected person/place so he can make a decision on whether affected employee/employees should come to work; ⚬ Each location organization can be given a form to send to their staff, customers, or visitors informing them of the probable contamination, and of safe and clean places.

**Table 2:** An Example of *OGC STA* Modelling for Various Data Streams for the Deployed *IoCT.*

Thing	Datastream			Sensor		ObservedProperty		Observation		FeatureOfInterest	
	Name	Description	Observation Type	Name	Description	Name	Description	Result	Phenomentime	Name	Description
IndoorGML cell name: Room326	Duration	Duration spent closed by the Thing	OM_Count Observation	Smartphone	Galaxy S9+	Time	Total time user was in the *Thing* in seconds	300	2020-06-08T23:14:09.438Z	IndoorGML cell name: Room326	Meeting Room 3rd floor
	Beacon Proximity	The ID broadcasted by the closest beacon	OM_ Observation	Smartphone	Galaxy S9+	Beacon ID	The beacon ID for the closest beacon	“uBFLQX2nXc5D3cS1”			
	Activity Type	The type of activity that was chosen by the user	OM_ Observation	Smartphone	Galaxy S9+	Activity Type	The type of activity that was chosen by the user	Cleaning			
	Entrance Time	The time that the user enters the proximity zone of the beacon	OM_ Observation	Smartphone	Galaxy S9+	Entrance Time	The time that the user enters the proximity zone of the beacon	2020-06-08T23:09:09.438Z			
	Cough Audio	The number of coughs detected by using the AI sound subsystem	OM_Count Observation	SunFounder Mini Microphone	SunFounder USB2.0 Mini Mic for Raspberry Pi4 Model B	Coughs Audio	The number of coughs detected by the audio subsystem	3			
	Cough Video	The number of coughs detected by the AI video subsystem	OM_Count Observation	Raspberry Pi Camera Module V2	Raspberry Pi Camera Module V2 connected to Jetson Xavier NX	Coughs Video	The number of coughs detected by the AI video subsystem	3			
	Video Proximity	The number of social distance violations detected by the AI video subsystem	OM_Count Observation	Raspberry Pi Camera Module V2	Raspberry Pi Camera Module V2 connected to Jetson Xavier NX	Violation Count	The number of social distance violations detected by the AI video subsystem	4			
	Video Touch	The number of touches detected by the AI video subsystem	OM_Count Observation	Raspberry Pi Camera Module V2	Raspberry Pi Camera Module V2 connected to Jetson Xavier NX	Touch Count	The number of touches detected by the AI video subsystem	2			

**Table 3:** Configuration Parameter for Risky Behavior Detection Model.

Parameter	Value
Iteration Rate	0.0001
Network Size	480 × 480
Number of Iterations	4000
Filter	21

**Table 4:** Evaluation Metrics for Evaluating the Video-Based Risky Behavior Detection.

Evaluation Measures	Equations	Description
Precision	$Precision=\frac{1}{n}\;\underset{j}{\sum }\frac{\left|R_{j}\;\cap^{}M_{j}\;\right|}{\left|R_{j}\right|}$ (3)	This is defined as the ratio of the total number of items appearing in both $R_{j}$ and $M_{j}$ to the total number of $R_{j}$ [66]. n is the total number of users. A higher value for the Precision means better performance and higher accuracy.
Recall	$Recall=\frac{1}{n}\;\underset{j}{\sum }\frac{\left|R_{j}\;\cap^{}M_{j}\;\right|}{\left|M_{j}\right|}$ (4)	The Recall measure is defined as the ratio of the total number of items appearing in both $R_{j}$ and $M_{j}$ to the number of $M_{j}$ [66]. Similarly, to the Precision measure, a higher value for Recall means a better performance and higher accuracy for the recommender algorithm.
F-Score	F Score = 2*(Recall * Precision)/(Recall + Precision) (5)	The F-score combines both the precision and recall into one metric that captures both properties. In other words, it is the weighted average for precision and recall. This metric gives an overview of the model results.

**Table 5:** Evaluating Precision, Recall, F-Score, and Number of Samples for Each Behavior Action Class.

Detected Activities	Precision	Recall	F-Score	Number of Samples for Transfer Learning
Person Count	0.77	0.91	0.83	834
Doorknob	0.89	0.73	0.80	621
Touching with Hand	0.82	0.71	0.76	633
Coughing	0.84	0.82	0.83	603
Hugging	0.96	0.61	0.74	634
Hand Shaking	0.73	0.58	0.78	608

Supplementary Materials includes a demo video showing the results of the smart camera deep learning detection algorithm.

**Table 6:** Summary of the VGG16 Architecture.

Layer		Feature Map	Size	Kernel Size	Stride	Activation
Input	Image	1	224 × 224 × 3	-	-	-
1	2 × Convolution	64	224 × 224 × 64	3 × 3	1	Relu ^1^
	Max Pooling	64	112 × 112 × 64	3 × 3	2	Relu
3	2 × Convolution	128	112 × 112 × 128	3 × 3	1	Relu
	Max Pooling	128	56 × 56 × 128	3 × 3	2	Relu
5	2 × Convolution	256	56 × 56 × 256	3 × 3	1	Relu
	Max Pooling	256	28 × 28 × 256	3 × 3	2	Relu
7	3 × Convolution	512	28 × 28 × 512	3 × 3	1	Relu
	Max Pooling	512	14 × 14 × 512	3 × 3	2	Relu
10	3 × Convolution	512	14 × 14 × 512	3 × 3	1	Relu
	Max Pooling Operator	512	7 × 7 × 512	3 × 3	2	Relu
13	FC ^2^	-	25,088	-	-	Relu
14	FC	-	4096	-	-	Relu
15	FC	-	4096	-	-	Relu
Output	FC	-	1000	-	-	Softmax

^1^ Rectified Linear Unit (ReLU) is a non-linear activation function. Max pooling layer applies a max pooling operation to its inputs. ^2^ Fully Connected (FC) layers follow a stack of convolutional layers with different depth.

**Table 7:** Evaluating Precision, Recall, F-Score, and Number of Samples for Audio-Based Detection.

	Precision	Recall	F-Score	No. of Samples for Transfer Learning
Coughing and Sneezing	0.77	0.91	0.83	3293
Coughing and Sneezing (Other Classes)	0.89	0.73	0.80	2536

**Table 8:** Performance of Different Functionalities on Various Platforms.

Performance (Millisecond)	BLE Proximity Detection	Video-Based People Density	Video-Based Physical Distancing	Video-Based Risky Behavior Detection	Audio-Based Risky Behavior Detection
Desktop	-	57	52	40	170
Jetson NX	-	670	590	250	230
Mobile App on Galaxy S9	<60	-	-	-	-

## Data Availability

Please refer to suggested Data Availability Statements in section “MDPI Research Data Policies” at https://www.mdpi.com/ethics.

## Supplementary Materials

The following are available online at https://www.mdpi.com/1424-8220/21/1/50/s1.

## References

[1] Whitelaw S., Mamas M.A.A., Topol E., Van Spall H.G. (2020). Applications of digital technology in COVID-19 pandemic planning and response. Lancet Digit. Health.

[2] Agrawal A., Gans J., Goldfarb A., Lederman M. (2020). The CEO’s guide to safely reopening the workplace. MIT Technol. Rev. https://www.technologyreview.com/2020/05/28/1002326/business-workplace-reopening-safely-testing-covid-19/.

[3] Wu Y., Lee T. Enhancing Sound Texture in CNN-based Acoustic Scene Classification. Proceedings of the ICASSP 2019—2019 IEEE International Conference on Acoustics, Speech and Signal Processing (ICASSP).

[4] Guo Z.-D., Wang Z.-Y., Zhang S.-F., Li X., Li L., Li C., Cui Y., Fu R.-B., Dong Y.-Z., Chi X.-Y. (2020). Aerosol and Surface Distribution of Severe Acute Respiratory Syndrome Coronavirus 2 in Hospital Wards, Wuhan, China, 2020. Emerg. Infect. Dis..

[5] Petrovic N. Simulation Environment for Optimal Resource Planning During COVID-19 Crisis. Proceedings of the 2020 55th International Scientific Conference on Information, Communication and Energy Systems and Technologies (ICEST).

[6] Berke l., Bakker M., Vepakomma P., Larson K., Pentland A.S. (2020). Assessing disease exposure risk with location data: A proposal for cryptographic preservation of privacy. arXiv.

[7] Kumar K., Kumar N., Shah R. (2020). Role of IoT to avoid spreading of COVID-19. Int. J. Intell. Networks.

[8] Mohammed M.N., Syamsudin H., Al-Zubaidi S., Sairah A.K., Ramli R., Yusuf E. (2020). Novel covid-19 detection and diagnosis system using IoT based smart helmet. Int. J. Psychosoc. Rehabil..

[9] Singh R.P., Javaid M., Haleem A., Suman R. (2020). Internet of things (IoT) applications to fight against COVID-19 pandemic. Diabetes Metab. Syndr. Clin. Res. Rev..

[10] Cheng H.-Y., Jian S.-W., Liu D.-P., Ng T.-C., Huang W.-T., Lin H.-H. (2020). For the Taiwan COVID-19 Outbreak Investigation Team. Contact Tracing Assessment of COVID-19 Transmission Dynamics in Taiwan and Risk at Different Exposure Periods before and After Symptom Onset. JAMA Intern. Med..

[11] Unified Research on Privacy-Preserving Contact Tracing and Exposure Notification for COVID-19. https://bit.ly/virustrackertracker.

[12] Stanley J., Granick J.S. (2020). Aclu White Paper: The Limits of Location Tracking in An Epidemic. Aclu White Paper. https://www.aclu.org/report/aclu-white-paper-limits-location-tracking-epidemic.

[13] Cho H., Ippolito D., Yu Y.W. (2020). Contact Tracing Mobile Apps for COVID-19: Privacy Considerations and Related Trade-offs. arXiv.

[14] Ahmed N., Michelin R.A., Xue W., Ruj S., Malaney R., Kanhere S.S., Seneviratne A., Hu W., Janicke H., Jha S.K. (2020). A Survey of COVID-19 Contact Tracing Apps. IEEE Access.

[15] Michael K., Abbas R. (2020). Behind COVID-19 Contact Trace Apps: The Google–Apple Partnership. IEEE Consum. Electron. Mag..

[16] Yu T., Jin H., Nahrstedt K. (2019). ShoesLoc: In-Shoe Force Sensor-Based Indoor Walking Path Tracking. Proc. ACM Interact. Mob. Wearable Ubiquitous Technol..

[17] Becker J.K., Li D., Starobinski D. (2019). Tracking Anonymized Bluetooth Devices. Proc. Priv. Enhancing Technol. Walter Gruyter GmbH.

[18] Dar A.B., Lone A.H., Zahoor S., Khan A.A., Naaz R. (2020). Applicability of mobile contact tracing in fighting pandemic (COVID-19): Issues, challenges and solutions. Comput. Sci. Rev..

[19] Kang H.K., Li K.J. (2017). A Standard Indoor Spatial Data Model—OGC IndoorGML and Implementation Approaches. ISPRS Int. J. Geo-Inf..

[20] Li K.J., Conti G., Konstantinidis E., Zlatanova S., Bamidis P. (2019). OGC IndoorGML: A standard approach for indoor maps. Geographical and Fingerprinting Data to Create Systems for Indoor Positioning and Indoor/Outdoor Navigation.

[21] OGC http://www.opengeospatial.org.

[22] OGC SensorThing API. http://ogc-iot.github.io/ogc-iot-api.

[23] Liang S.H., Croitoru A., Tao C.V. (2005). A distributed geospatial infrastructure for Sensor Web. Comput. Geosci..

[24] CCIT University of Calgary Campus Map. https://asc.ucalgary.ca/building/calgary-centre-for-innovative-technology/.

[25] Worldometers Worldometers Post on COVID-19 Coronavirus/Transmission. https://www.worldometers.info/coronavirus/transmission/.

[26] He P. Study on Epidemic Prevention and Control Strategy of COVID-19 Based on Personnel Flow Prediction. Proceedings of the 2020 International Conference on Urban Engineering and Management Science (ICUEMS).

[27] World Health Organization WHO Guidelines for the Production, Control and Regulation of Snake Antivenom Immunoglobulins. http://www.who.int/bloodproducts/snake_antivenoms/snakeantivenomguide/en/.

[28] https://www.canada.ca/en/public-health/services/diseases/2019-novel-coronavirus-infection.html?&utm_campaign=gc-hc-sc-coronavirus2021-ao-2021-0005-9834796012&utm_medium=search&utm_source=google_grant-ads-107802327544&utm_content=text-en-434601690164&utm_t.

[29] Liang S.H.L., Huang C.-Y. (2013). GeoCENS: A Geospatial Cyberinfrastructure for the World-Wide Sensor Web. Sensors.

[30] Luo K., Saeedi S., Badger J., Liang S. (2018). Using the Internet of Things to Monitor Human and Animal Uses of Industrial Linear Features. Comput. Vis. ECCV 2020.

[31] Jazayeri M.A., Liang S.H.L., Huang C.-Y. (2015). Implementation and Evaluation of Four Interoperable Open Standards for the Internet of Things. Sensors.

[32] Saeedi S., Luo K., Badger J., Liang S. (2017). Using OGC SensorThings API for Boreal Environmental Monitoring. In Spatial Knowledge and Information, Banff, Canada. http://beraproject.org/wp-content/uploads/2016/12/SSaedi_BERAPoster.pdf.

[33] Lieberman J., Liang S., Hawkins B., Chen C., Starkov I., MacDonald J., Alzona M., Botts M.J.M., Saeedi S. (2019). OGC SCIRA Pilot Engineering Report.

[34] Schumann G., Lieberman J., Jirka S., Alzona M., Alamdar F., Botts M., Brackin R., Clark C., Galiber F., Kalantari M. (2018). Incident Management Information Sharing (IMIS) Internet of Things (IoT) Architecture Engineering Report.

[35] Liang S.H. (2017). Sensor Networks, The Sensor Web, and the Internet of Things. Int. Encycl. Geogr. People Earth Environ. Technol..

[36] Chen C., Hao C., Jeong H., Lieberman J., Li K., Stark L., Nishesh R., Liang S., Braun T., Santhanavanich T. (2020). OGC 3D-IoT Platform for Smart Cities Engineering Report.

[37] Trilles S., Lujan A., Belmonte-Fernández Ó., Montoliu R., Torres-Sospedra J., Huerta J. (2015). SEnviro: A Sensorized Platform Proposal Using Open Hardware and Open Standards. Sensors.

[38] Liang S.H.L., Khalafbeigi T. (2019). OGC SensorThings API Part 2–Tasking Core, Version 1.0..

[39] OASIS OASIS MQTT 3.1.1 Specification. http://docs.oasis-open.org/mqtt/mqtt/v3.1.1/mqtt-v3.1.1.html.

[40] (2016). OGC SensorThings API Part I: Sensing. https://docs.opengeospatial.org/is/15-078r6/15-078r6.html.

[41] ISO 19156:2011 Geographic information—Observations and measurements. International Organization for Standardization..

[42] Dutta A., Sameer S., Kumar A.S. (2017). Development of CityGML Application Domain Extension for Indoor Routing and Positioning. J. Indian Soc. Remote. Sens..

[43] Ryoo H.G., Kim T., Li K.-J. Comparison between Two OGC standards for indoor space: CityGML and indoorGML. Proceedings of the Seventh ACM SIGSPATIAL International Workshop on Indoor Spatial Awareness.

[44] Gunduz M., Isikdag U., Basaraner M. (2016). A Review of Recent Research in Indoor Modelling & Mapping. ISPRS Int. Arch. Photogramm. Remote. Sens. Spat. Inf. Sci..

[45] OGC (2012). OGC City Geography Markup Language (CityGML) Encoding Standard 2.0.0, Standard OGC 12-019. https://www.ogc.org/standards/citygml.

[46] OGC (2017). OGC® IndoorGML 1.0.3, Standard OGC 14-005r5. https://www.ogc.org/standards/indoorgml.

[47] (2018). I. 16739-1:2018. Industry Foundation Classes (IFC) for Data Sharing in the Construction and Facility Management Industries International Standard. https://www.iso.org/standard/51622.html.

[48] Tekavec J., Lisec A. (2020). Cadastral data as a source for 3D indoor modelling. Land Use Policy.

[49] NVIDIA (2020). Jetson Xavier NX Developer Kit. https://developer.nvidia.com/embedded/jetson-xavier-nx-devkit.

[50] Saeedi S., Malek M.R., Delavar M.R., Tayyebi A., Ruan D., Montero J., Lu J., Martínez L., D’Hondt P., Ghent University (2008). An Intuitionistic Fuzzy Analytical Network Process for Parking Site Selection. Computational Intelligence in Decision and Control.

[51] Afshari A.R., Yusuff R.M. A review of Spatial Multi Criteria Decision Making. Proceedings of the 6th SAS Tech 2012.

[52] Fusade-Boyer M., Pato P.S., Komlan M., Dogno K., Batawui K., Go-Maro E., McKenzie P., Guinat C., Secula A., Paul M. (2020). Risk Mapping of Influenza D Virus Occurrence in Ruminants and Swine in Togo Using a Spatial Multicriteria Decision Analysis Approach. Viruses.

[53] Malczewski J., Jankowski P. (2020). Emerging trends and research frontiers in spatial multicriteria analysis. Int. J. Geogr. Inf. Sci..

[54] Van Doremalen N., Bushmaker T., Morris D.H., Holbrook M.G., Gamble A., Williamson B.N., Tamin A., Harcourt J.L., Thornburg N.J., Gerber S.I. (2020). Aerosol and surface stability of SARS-CoV-2 as compared with SARS-CoV-1. N. Engl. J. Med..

[55] Kampf G., Lemmen S., Suchomel M. (2020). Ct values and infectivity of SARS-CoV-2 on surfaces. Lancet Infect. Dis..

[56] Mondelli M.U., Colaneri M., Seminari E.M., Baldanti F., Bruno R. (2020). Low risk of SARS-CoV-2 transmission by fomites in real-life conditions. Lancet Infect. Dis..

[57] The Lancet Respiratory Medicine (2020). COVID-19 transmission—up in the air. Lancet Respir. Med..

[58] Morawska L., Cao J. (2020). Airborne transmission of SARS-CoV-2: The world should face the reality. Env. Int..

[59] IETF The GeoJSON Format (RFC 7946). https://tools.ietf.org/html/rfc7946.

[60] Montoliu R., Sansano E., Gascó A., Belmonte O., Caballer A. (2020). Indoor Positioning for Monitoring Older Adults at Home: Wi-Fi and BLE Technologies in Real Scenarios. Electronics.

[61] Ojagh S., Saeedi S., Liang S. (2020). A Person-to-Person and Person-to-Place COVID-19 Contact Tracing System Based on OGC IndoorGML. ISPRS Int. J. Geo-Inf..

[62] Lin T.Y., Maire M., Belongie S., Hays J., Perona P., Ramanan D., Dollár P., Zitnick C.L. (2014). Microsoft COCO: Common objects in context. Computer Vision—ECCV 2014. ECCV 2014. Lecture Notes in Computer Science.

[63] Ji H., Zeng X., Li H., Ding W., Nie X., Zhang Y., Xiao Z. Human abnormal behavior detection method based on T-TINY-YOLO. Proceedings of the 5th International Conference on Multimedia and Image Processing.

[64] Shinde S., Kothari A., Gupta V. (2018). YOLO based Human Action Recognition and Localization. Procedia Comput. Sci..

[65] Quattoni A., Collins M., Darrell T. Transfer learning for image classification with sparse prototype representations. Proceedings of the 2008 IEEE Conference on Computer Vision and Pattern Recognition.

[66] Goldberg K., Roeder T., Gupta D., Perkins C. (2001). Eigentaste: A Constant Time Collaborative Filtering Algorithm. Inf. Retr..

[67] Allen J. Applications of the short time Fourier transform to speech processing and spectral analysis. Proceedings of the 2015 IEEE International Conference on Acoustics, Speech and Signal Processing (ICASSP).

[68] Stevens S., Volkmann J., Newman E. (1937). A Scale for the Measurement of the Psychological Magnitude Pitch. J. Acoust. Soc. Am..

[69] Rioul O., Vetterli M. (1991). Wavelets and signal processing. IEEE Signal Process. Mag..

[70] Librosa Librosa.Feature.Spectral-Librosa 0.7.2 Documentation. https://librosa.org/doc/latest/index.html.

[71] Abeßer J. (2020). A Review of Deep Learning Based Methods for Acoustic Scene Classification. Appl. Sci..

[72] Takahashi N., Gygli M., Pfister B., Van Gool L. (2016). Deep convolutional neural networks and data augmentation for acoustic event detection. arXiv.

[73] Simonyan K., Zisserman A. Very deep convolutional networks for large-scale image recognition. Proceedings of the International Conference on Learning Representations.

[74] Qassim H., Verma A., Feinzimer D. Compressed residual-VGG16 CNN model for big data places image recognition. Proceedings of the 2018 IEEE 8th Annual Computing and Communication Workshop and Conference (CCWC).

[75] Zhang Z., Xu S., Cao S., Zhang S. (2018). Deep Convolutional Neural Network with Mixup for Environmental Sound Classification. Chinese Conference on Pattern Recognition and Computer Vision (PRCV).

[76] Shen S., Yang H., Sheng M. (2018). Compression of a Deep Competitive Network Based on Mutual Information for Underwater Acoustic Targets Recognition. Entropy.

[77] Piczak K. ESC: Dataset for Environmental Sound Classification. Proceedings of the 23rd ACM international Conference on Multimedia.

[78] Gemmeke J.F., Ellis D.P.W., Freedman D., Jansen A., Lawrence W., Moore R.C., Plakal M., Ritter M. Audio Set: An ontology and human-labeled dataset for audio events. Proceedings of the 2017 IEEE International Conference on Acoustics, Speech and Signal Processing (ICASSP).

[79] Ojagh S., Malek M.R., Saeedi S., Liang S.H.L. (2018). An Internet of Things (IoT) Approach for Automatic Context Detection. Proceedings of the 2018 IEEE 9th Annual Information Technology, Electronics and Mobile Communication Conference (IEMCON).

[80] Ojagh S., Malek M.R., Saeedi S. (2020). A Social–Aware Recommender System Based on User’s Personal Smart Devices. ISPRS Int. J. Geo-Inform..

