# Supplementary material for: An Interoperable Architecture for the Internet of COVID-19 Things (IoCT) Using Open Geospatial Standards—Case Study: Workplace Reopening
Source: Sensors (Basel). 2020 Dec 24;21(1):50. doi: 10.3390/s21010050 (PMC7796058; doi:10.3390/s21010050)
Supplement: Supplementary file 1 [file sensors-21-00050-s001.pdf]

1. OGC SensorThings API JSON Payloads

1.1 Smartphone JSON Payload

The JSON payload for pushing different observations gathered by smartphones using the OGC SensorThings API is shown in the following code block and accessible from this link: https://zenodo.org/record/4382148/files/Smartphone%20JSON%20Payload.txt?download=1

1.2. Jetson JSON Payload

The JSON payload for pushing different observations gathered by the Jetson Xavier NX Developer Kit to the instance of the OGC SensorThings API is shown in the following code block and accessible from this link:
https://zenodo.org/record/4382148/files/Jetson%20JSON%20Payload.txt?download=1

2. Cleaning Scenario

The demo videos related to implementing the cleaning scenario are presented in this section.

2.1. Room Cleaning

Video B1 shows the cleaner entering the room, setting the app status to clean, and cleaning the doorknob, then leaving the room. The person was also detected by the smart camera. https://zenodo.org/record/4382148/files/DV1.mp4?download=1

2.2. People Trajectories

Video B2 shows people and cleaners moving around the building and how the risk profile changes accordingly. https://zenodo.org/record/4382148/files/DV2.mp4?download=1

2.3. Physical Distancing

Video B3 shows the applied method for physical distancing in an outdoor setting. https://zenodo.org/record/4382148/files/DV3.mp4?download=1

2.4. Smart Camera

Video B4 shows the results of the smart camera deep learning detection algorithm for coughs and door touching. https://zenodo.org/record/4382148/files/DV4.mp4?download=1

2.5. Risk Evaluation Dashboard

Video B5 presents the risk profile of the room based on people density, risky behaviors, and cleaning activities. https://zenodo.org/record/4382148/files/DV5.mp4?download=1

2.6. Risk Evaluation Scenario (Merged Scenario)

Video B6 presents the risk profile of the room based on people density and risky behaviors (Supplementary materials 2.4) as well as cleaning activities (Supplementary materials 2.1). https://zenodo.org/record/4382148/files/DV6.mp4?download=1
